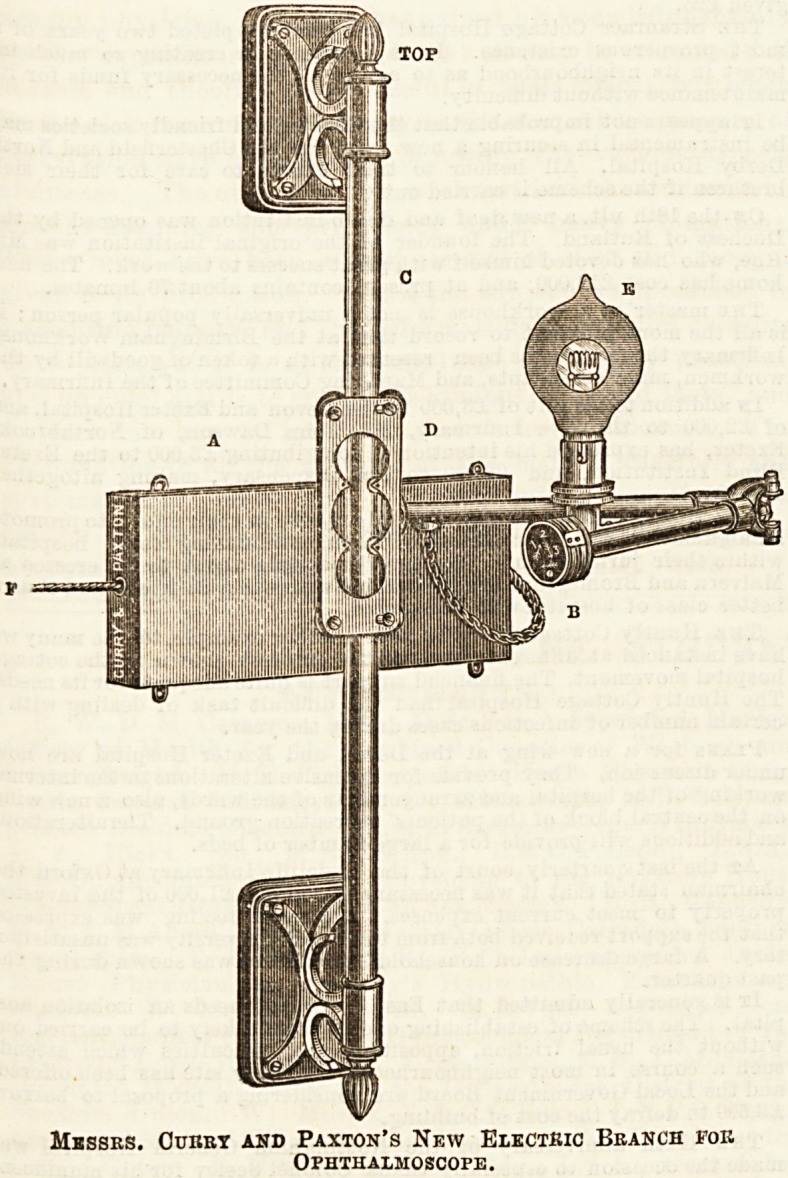# Electric Lighting for Hospitals

**Published:** 1894-11-17

**Authors:** 


### PRACTICAL DEPARTMENTS.

### ELECTRIC LIGHTING FOR HOSPITALS.

(*Continued from page* 105).

Although, as we have already mentioned, the Middlesex has so far been the only one of the large London general hospitals to adopt the system of electric lighting throughout the length and breadth of the building, in several a partial installation has been effected in the out-patient department and operating theatre, one or both.

At St. George's Hospital the experiment was first tried in the theatre, and then extended to the out-patient department and dispensary. The electric light has been laid on to this extent also at the Westminster Hospital. At these two institutions and at Guy's, where the theatre is at present the only portion of the building as yet thus illuminated, the work was carried out by Messrs. Drake and Gorham, Victoria Street, S.W. The attention of Mr. Drake was especially called to the question of electric lighting, as applied to institutional purposes, through an accident, which caused him to be carried as a patient into Westminster Hospital with a fractured thigh, and while thus a prisoner the realisation of the boon which an improved system of lighting would confer alike on patients, doctors, and nurses, both for safety and comfort, was brought forcibly before his notice. Though at present the wards at Westminster are not fitted with the electric light, there is, we understand, a standard lamp, which can be there used when required, and moved from bed to bed. The out-patient department is very thoroughly fitted up, and

Laryngoscope Lamp (Westminster Hospital).

by Miss Pyne's kind permission we here reproduce a sketch of the lamp there in use for laryngoscopic and ophthalmoscopic purposes.

There is not a great deal of difference in the special fittings used in the ophthalmic and throat and ear departments at the various hospitals which we have seen, but a new and

Messrs. Curry and Paxton's New Electric Branch for Ophthalmoscope.

improved branch for the ophthamoscope, which has been recently brought into use at Moorfields, deserves special notice. Messrs. Curry and Paxton, 195, Great Portland Street, are the introducers of this branch, of which, by their permission, we here give an illustration. It is fitted with an adjusting switch (B) to graduate the light in the same way that a gas-jet is graduated by a stop-cock, and an Edison and Swan's focus lamp, specially obscured to obliterate the filament, giving a solid disc of light. One side of the lamp is clear; it is therefore made to rotate, so as to admit of either side being used. The branch can be adapted to any installation by means of an ordinary plug, and is an important improvement upon any ophthalmoscope hitherto in use.

The new Royal Eye Hospital, Southwark, which is in many respects a type of an up-to-date institution, was fitted with electric light at the commencement of its history two years ago, but the special apparatus in the consulting-rooms require some improvement before they can compete with so perfect an arrangement as the one we have described above. They would be more satisfactory were they fitted with universal joints, enabling the position of the light to be more easily altered at the will of the surgeon. The improvements in such fittings follow one upon the other, as may be expected, and in this as in other respects more are still to be hoped for in the future.

## Figures and Tables

**Figure f1:**
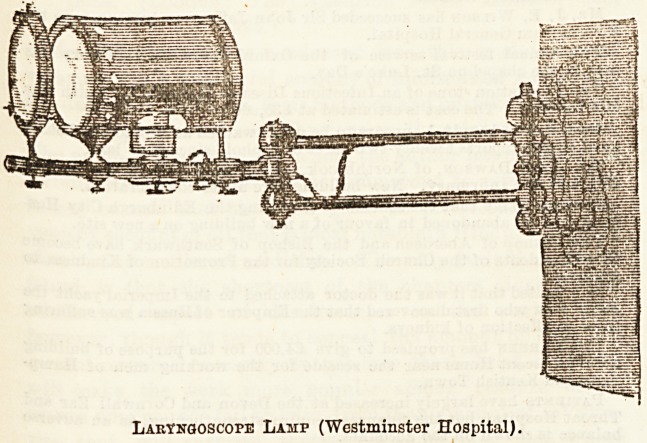


**Figure f2:**